# Functional Redox Proteomics Reveal That *Salvia miltiorrhiza* Aqueous Extract Alleviates Adriamycin-Induced Cardiomyopathy via Inhibiting ROS-Dependent Apoptosis

**DOI:** 10.1155/2020/5136934

**Published:** 2020-09-09

**Authors:** Yu-Chiang Hung, Pei-Wen Wang, Tung-Yi Lin, Pei-Ming Yang, Jyh-Sheng You, Tai-Long Pan

**Affiliations:** ^1^Department of Chinese Medicine, College of Medicine, Kaohsiung Chang Gung Memorial Hospital and Chang Gung University, Kaohsiung, Taiwan; ^2^Department of Medical Research, China Medical University Hospital, China Medical University, Taichung, Taiwan; ^3^Department of Traditional Chinese Medicine, Chang Gung Memorial Hospital, Keelung, Taiwan; ^4^TMU Research Center of Cancer Translational Medicine, Taipei Medical University, Taipei, Taiwan; ^5^Graduate Institute of Cancer Biology and Drug Discovery, College of Medical Science and Technology, Taipei Medical University, Taipei, Taiwan; ^6^School of Traditional Chinese Medicine, Chang Gung University, Taoyuan, Taiwan; ^7^Liver Research Center, Chang Gung Memorial Hospital, Taoyuan, Taiwan; ^8^Research Center for Chinese Herbal Medicine and Research Center for Food and Cosmetic Safety, College of Human Ecology, Chang Gung University of Science and Technology, Taoyuan, Taiwan

## Abstract

The anticancer agent adriamycin (ADR) has long been recognized to induce a dose-limiting cardiotoxicity, while *Salvia miltiorrhiza* (SM) is a Chinese herb widely used for the treatment of cardiovascular disorders and its aqueous extract (SMAE) has shown anticancer as well as antioxidant effects. In the current study, we aimed at investigating the synergistic effect and potent molecular mechanisms of SMAE with a focus on the cardioprotective benefit observed under ADR adoption. Histopathological analysis indicated that SMAE could substantially alleviate cardiomyopathy and cell apoptosis caused by ADR. Meanwhile, the two-dimensional electrophoresis (2-DE) oxyblots demonstrated that SMAE treatment could effectively reduce carbonylation of specific proteins associated with oxidative stress response and various metabolic pathways in the presence of ADR. SMAE application also showed protective efficacy against ADR-mediated H9c2 cell death in a dose-dependent manner without causing any cytotoxicity and significantly attenuated the reactive oxygen species production. Particularly, the simultaneous administration of ADR and SMAE could remarkably suppress the growth of breast cancer cells. We also noticed that there was a marked upregulation of detoxifying enzyme system in the presence of SMAE, and its exposure also contributed to an increase in Nrf2 and HO-1 content as well. SMAE also amended the ERK/p53/Bcl-xL/caspase-3 signaling pathways and the mitochondrial dysfunction, which eventually attribute to apoptotic cathepsin B/AIF cascades. Correspondingly, both the ERK1/2 inhibitor (U0126) and pan-caspase inhibitor (Z-VAD-FMK) could at least partially abolish the ADR-associated cytotoxicity in H9c2 cells. Collectively, these results support that ROS apoptosis-inducing molecule release is closely involved in ADR-induced cardiotoxicity while SMAE could prevent or mitigate the causative cardiomyopathy through controlling multiple targets without compromising the efficacy of chemotherapy.

## 1. Introduction

The anthracycline antibiotic adriamycin (ADR), also known as doxorubicin, is the commonly used chemotherapeutic agent for the treatment of various cancers. However, the severe cardiotoxicity of ADR greatly restricts its clinical utilization [1, 2]. Although ADR-related injury appears to be multifactorial and complicated, oxidative stress and mitochondrial dysfunction have been proposed to majorly account for the pathogenesis of ADR-induced cardiomyopathy [3–6]. Moreover, cardiomyocytes apoptosis can be a pivotal event by which ADR causes deterioration of cardiac system but the detailed molecular mechanisms still remain unclear. Several chemical agents have been used to prevent the ADR-induced cardiotoxicity; nevertheless, treatment with these drugs is known to have certain disadvantages. Dexrazoxane (DXR), for example, is the only FDA-approved drug for treating ADR-associated cardiomyopathy; however, it might increase the risk of infection, myelosuppression, and second primary malignancies [7, 8]. Therefore, the development of alternative cardioprotectants with low toxicity is urgently needed for cancer survivors.

A great amount of effort has been made towards perfecting the clinical applications of herbal medicine that are frequently used in combination to improve the therapeutic efficacy [9]. Of note, *Salvia miltiorrhiza* (SM) has been prescribed for treating cardiovascular disorders since a long time [10]. The water-soluble components extracted from SM including salvianolic acids A, B, and C have garnered much interest as a multitargeted therapy owing to their antioxidant effects on free radicals. Additionally, *Salvia miltiorrhiza* aqueous extract (SMAE) has been utilized widely for the treatment of coronary heart disease, atherosclerosis, and ischemic cardiovascular diseases [11, 12]. Our previous research has also revealed that SMAE provides a protective effect against adriamycin- (ADR-) induced cardiomyopathy and hepatic damage [13]. However, the molecular pathways associated with the SMAE-mediated advantageous effect in managing the cardiotoxicity resulting from ADR treatment have not been thoroughly investigated. Herein, we used animal and cellular models to verify whether ADR-induced cardiomyopathy could be relieved by SMAE and also evaluate the synergistic effect on cancer cells.

ROS generation caused by ADR administration would disturb the normal redox balance and produce huge oxidative stress, which subsequently stimulates the carbonylation of specific groups of proteins involved in physiological dysfunction and induces cardiomyocyte apoptosis [14–16]. The most largely studied oxidative stress–caused by a modification of proteins is the formation of carbonyl groups which can react with 2,4-dinitrophenylhydrazine (DNP) and are discovered by redox proteomics [17, 18]. The appearance of carbonylated proteins has been considered as a hallmark of ROS-induced change of protein which might be helpful to predict novel targets for the diagnosis and prognosis of diseases. Furthermore, the functional “signature network” analyzed with MetaCore™ pathway generates global cellular mechanisms underlying different protein levels and imitates signaling pathways based on the integration of molecular and clinical information [19].

The present study has shed light on the cellular mechanisms involved with SMAE, exhibiting its protective effect and inhibition of ADR-induced cardiomyopathy. Additionally, our findings should offer an opportunity to execute novel therapeutic strategies and sensitive markers for the clinical usage of ADR.

## 2. Materials and Methods

### 2.1. Materials

Specific antibodies to GAPDH, SOD, catalase, p53, Nrf2, and HO-1 were purchased from Santa Cruz (Santa Cruz, CA, USA). Monoclonal antibodies to *β*-actin were obtained from Millipore (Burlingame, CA, USA). Polyclonal antibodies to caspase-3, -9, PARP, Bax, Bcl-xL, phosphor-p53, ERK, and phospho-ERK were purchased from Cell Signaling (Beverly, MA, USA). U0126 and Z-VAD-FMK were obtained from Enzo Life Science (New York, NY, USA).

### 2.2. Prepare the Extract of Salvia miltiorrhiza

The air-dried roots of cultivated *Salvia miltiorrhiza* Bunge (Labiatae) were purchased from a Traditional Chinese Medicine dispensary in Taiwan and authenticated by the experts in pharmacognosy. The preparation of *Salvia miltiorrhiza* aqueous extract (SMAE) was as reported by the method of Liu [20]. Briefly, the hot-water extract was prepared by boiling the dried roots with distilled water for 5 hr. The concentration used in each experiment was calculated upon the dry weight of the SMAE extract (mg/mL), which was resuspended in freshly prepared double distilled and deionized water as purified by the Milli-Q filtration system (Millipore).

### 2.3. ADR-Induced Heart Failure on Animal Model

Male Wistar rats, body weight 250–300 g, were maintained on a normal rat chow diet. The rats were randomly divided into three groups: CTL, ADR, and ADR plus SMAE. Five rats that received ready-to-use ADR (Adriblastina RD 10 mg-INS-040731, Pfizer Inc.) were administered by intraperitoneal injection in six equal doses (each containing 3 mg/kg ADR) over a period of 2 weeks, with a total cumulative dose of 18 mg/kg ADR, and five CTL rats were injected with an identical volume of normal saline. Five rats were administrated with 100 mg/kg/day of SMAE via oral delivery with ADR injections. At the end of the 5-week posttreatment period, the heart was removed and separately stored for histomorphologic examination and protein expression analysis [13]. Thereby, we utilized the short-term model to evaluate cardiotoxicity, while a previous study had shown that 10 mg/kg ADR application would result in 80% mortality [21], which was consistent with our findings. The rats were treated according to the Ethical Guidelines of the Animal Center, and the experimental protocol was reviewed and approved by the Institutional Animal Care and Use Committee of Chang Gung University (CGU08-61).

### 2.4. Histology and Immunohistochemistry

The heart tissue fixed by 5% neutral buffered formalin was immersed in paraffin and then sliced into 5 *μ*m sections. The sample slices were stained with Masson's trichrome (MT) for a histological assessment. Immunohistochemistry with caspase 3 was applied to specimens as previously described [22]. The histological changes were observed by using optical microscopy (Olympus BX51, Japan) in nonconsecutive, randomly chosen 400× histological fields. The digital photomicrographs were then processed with DP-72.

### 2.5. TUNEL Assay

Apoptosis was assessed by terminal deoxynucleotidyl transferase- mediated dUTP biotin nick end labeling (TUNEL) using ApopTag® Plus Peroxidase in situ Apoptosis Detection Kit (Millipore) according to the manufacturer's instructions. The slides were counterstained with hematoxylin and mounted. The numbers of stained and unstained cells were then counted from randomly chosen fields per slide within a high-power field (×400 magnifications) under an Olympus BX50 microscope [22].

### 2.6. Two-Dimensional Polyacrylamide Gel Electrophoresis (2-D PAGE)

The procedure has been reported previously [23]. Proteins (150 *μ*g for 2-DE oxyblot or 250 *μ*g for silver stain) were solubilized in the rehydrated buffer and applied onto 13 or 18 cm Immobiline DryStrip 3-10NL on the IPGphor IEF System (GE Healthcare). The running conditions of the IEF (isoelectric focusing) follow 30 V, 12 h; 100 V, 1 h; 250 V, 1 h; 500 V, 0.5 h; 1,000 V, 0.5 h; 4,000 V, 0.5 h; and 8,000 V, up to 80 kVh.

### 2.7. Derivatization of Protein Carbonyls and DNP Immunostaining

IPG strips were incubated in 2 N HCl with 10 mM DNPH at 25°C for 15 min after IEF; strips were then washed with 2 M Tris-Base/30% glycerol for 15 min [24]. The IPG strips were used for molecular weight-dependent separation of proteins by SDS-PAGE and transferred the protein blotting to a membrane which was incubated overnight at 4°C with the anti-DNP antibody in TBST containing 5% milk. The blots were washed and incubated goat anti-rabbit IgG HRP conjugate for 2 hrs. Enhanced chemiluminescence (PerkinElmer, CA, USA) was used for detection.

### 2.8. In-Gel Enzymatic Digestion and Mass Spectrometry

Spots of interest were excised and in-gel digested with trypsin according to previously described procedures [25]. Monoisotopic peptide masses were assigned and used for database searches with the MASCOT search engine (http://www.matrixscience.com) (Matrix Science, London). Search parameters were set as follows: a maximum allowed peptide mass error of 50 ppm and consideration of one incomplete cleavage per peptide.

### 2.9. Biological Network Analysis Using MetaCore™

Apply MetaCore™ software (vers. 5.2 build 17389, GeneGo, St. Joseph, MI, USA) to reveal associated ontological classes and relevant pathways, which were represented among the proteins identified by the 2-DE and peptide mass fingerprint [25].

### 2.10. Cell Culture and MTT Assay

Rat cardiomyoblast-derived H9c2 cell was purchased from the Food Industry Research and Development Institute. The H9c2 and MCF-7 cells were maintained in DMEM medium containing 10% fetal bovine serum (FBS) at 37°C in a humidified atmosphere of 5% CO^2^. Cell viability was determined by MTT. A total of 1 × 10^4^ cells were seeded in 24-well plates for 24 hours (h) and made quiescent by incubating in medium containing 0.2% FBS overnight. After treating with various concentrations (0, 0.3125, 0.625, 1.25, 2.5, 5, and 10 mg/mL) of SMAE for 48 h, isopropanol solution mixed with tetrazolium salt was added to the wells and incubated for additional 4 h at 37°C [26]. The optical density of the dissolved material was measured spectrophotometrically at 570 nm, and assays were performed in triplicate.

### 2.11. Image Analysis for Generation of Intracellular ROS under SMAE Application

2 × 10^3^ H9c2 cells were seeded in a slide chamber, grown to 60% confluence, and cultured in serum-free DMEM medium overnight. Cells were then incubated with 2.5 mg/mL of SMAE for 6 h. Carboxy-H2DCFDA (4 *μ*M, dissolved in PBS) was added to the wells and incubated for 30 min at 37°C. To terminate the reaction, the cells were washed with PBS twice. Next, 500 *μ*L culture medium was added to each well and incubated for 20 min at 37°C. The cells were observed and photographed using a fluorescent microscope (Olympus BX51) under the DP72 PhotoImage system. Image-Pro® plus 4.5 (Media Cybernetics, Bethesda, MD) image analysis software was used to quantify image signals [27].

### 2.12. Western Blot Analysis

Samples proteins were isolated by cell lysis buffer (Cell Signaling, MA, USA) and measured using the Bradford Protein Assay Kit (AMRESCO, OH, USA). Total proteins were separated with 10% SDS-polyacrylamide gel electrophoresis and transferred to PVDF membrane (PALL). Next, the blots were incubated with specific primary antibody overnight at 4°C after blocking and further incubated with a peroxidase-labeled anti-mice or -rabbit IgG for 2 h; blots were then washed and incubated goat anti-rabbit and anti-mouse IgG (Chemicon) HRP conjugate for 2 hrs. Enhanced chemiluminescence (PerkinElmer, USA) was used for signal detection. The level of expression of *β*–actin was used as a gel loading control [27].

### 2.13. Statistical Analysis

All values were presented as the mean ± SD. Statistical analysis of the mean values was carried out with the ANOVA test using SPSS software (SPSS Inc., Chicago, IL, USA). Differences were considered as being significant at *p* < 0.05.

## 3. Results

### 3.1. Effects of SMAE on the Pathological Characteristics, Caspase-3 Level, and Apoptosis in ADR-Exposed Cardiomyocyte of Rats

**Figure 1:**
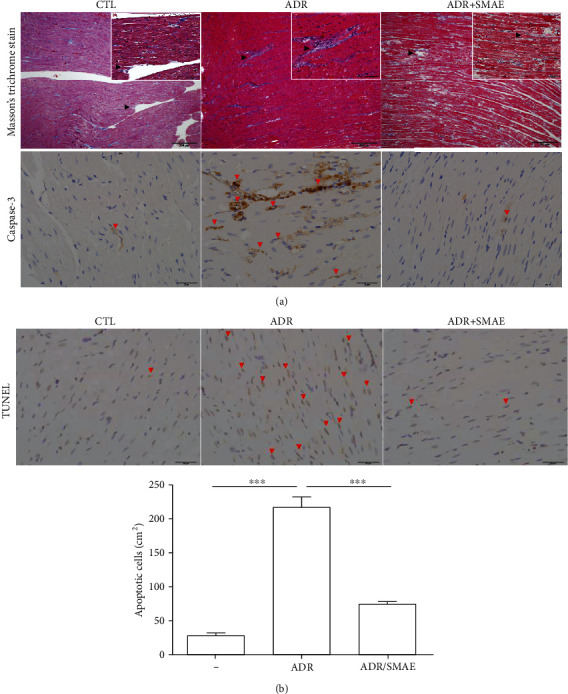
(a) Upper panels: histologic examination of heart tissue by Masson's trichrome staining and immunohistochemical analysis for control group, ADR-treated group, and ADR+SMAE group. The positive signals showing blue color demonstrated an accumulation of collagen and were zoomed in by white squares (indicated by black arrows; original magnification: ×200). Lower panels: the regions with differently expressed caspase-3 were indicated by red arrows. Original magnification: ×400. (b) Terminal deoxynucleotidyl transferase dUTP nick end labeling (TUNEL) experiment was performed with immunohistochemical examination of rat heart, and the red arrows indicated TUNEL-positive signals. The quantification of the TUNEL-positive cardiomyocytes per cm^2^ in various treatments was shown by bar chart. Data are mean ± SD (^∗∗∗^*p* < 0.001).

Drug-caused cardiotoxicity has become a critical issue linked to the therapeutic efficiency. Given that the protective effects of SMAE were evaluated by the extent of collagen accumulation and death of cardiomyocyte *in vivo*. As shown in Figure 1(a), ADR-applied hearts showed moderate damage characterized with collagen accumulation as detected by the use of Masson's trichrome staining, while the morphology of control tissue remained normal. Conversely, the rats which were simultaneously subjected to SMAE and ADR treatment presented normal heart histological characteristics, and no sign of cardiac fibrogenesis was detected. Furthermore, cardiomyocytes apoptosis as a consequence of ADR exposure was also evaluated, which was characterized by the presence of altered heart foci. The immunohistochemistry examination showed that they stained positively for caspase-3. In contrast, caspase-3 signal was rarely identified in samples treated with SMAE. Meanwhile, the terminal deoxynucleotidyl transferase-mediated dUTP biotin nick end labeling (TUNEL) results showed significantly increased cell death in the ADR-applied samples compared with the control; reversely, a minor apoptotic signal was detected in the ADR/SMAE-treated group (Figure 1(b)), implying a strong protection of SMAE against ADR-induced cardiocytotoxicity.

### 3.2. Detection of the Protein Carbonylation with 2-DE Oxyblot and Functional Network Analysis

**Figure 2:**
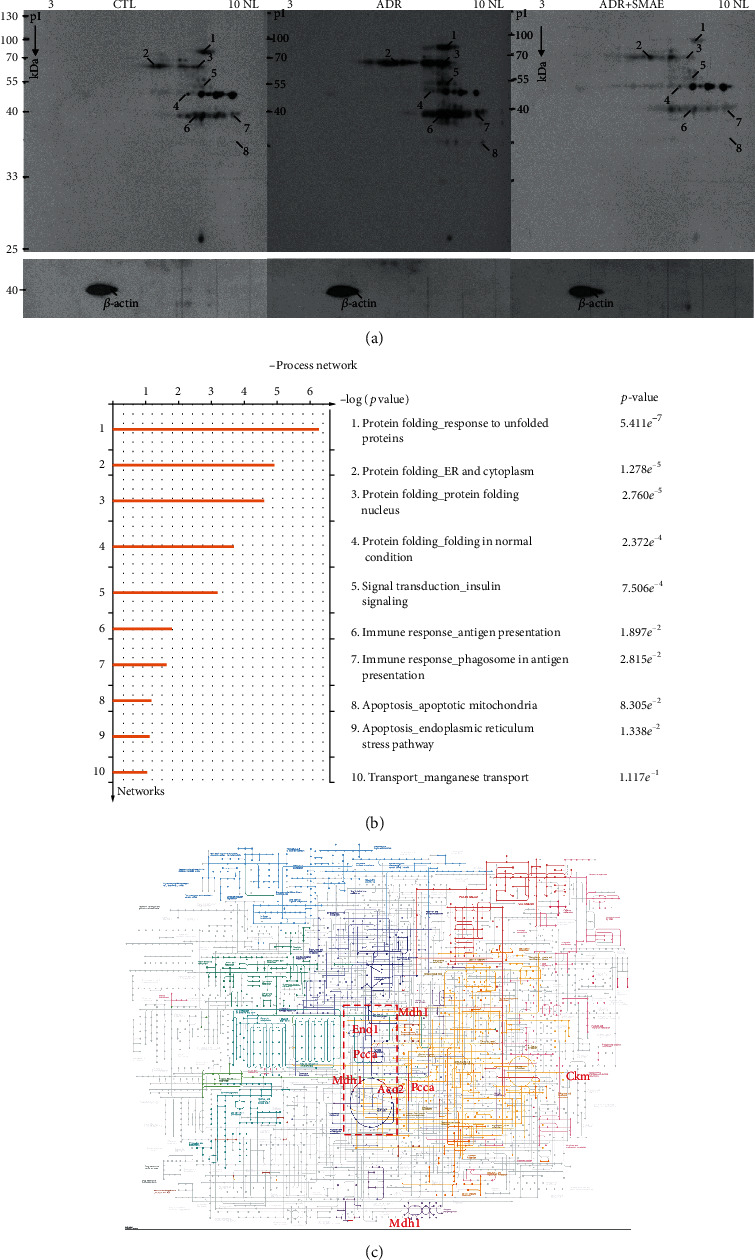
(a) Images of the 2-DE oxyblot. Analysis of protein oxidation levels in DNP-derivatized cellular proteins among the control group, ADR-treated group, and ADR+SMAE group. A significant reduction in the carbonylation levels of proteins are identified in the ADR+SMAE-applied group compared to the ADR group. (b) Top-ranked pathways from the GeneGo MetaCore™ pathway analysis. Pathways were ranked according to *p* values, and bars represent the inverse log of the *p* value. (c) The KEGG analysis indicated that several metabolisms processed in mitochondria were affected under the ADR treatments.

Oxidative modification of proteins resulting from ADR application has been implicated as one of the leading causes of cardiac cell death. Meanwhile, carbonylation of proteins due to oxidative modification has been reported to impair their normal function in various metabolic processes. In our present study, changes in oxidized proteins among different groups were delineated by 2-DE oxyblots. The extent of protein oxidation was dramatically upregulated in the ADR group with respect to the control sample. Interestingly, there was a remarkably decreasing tendency for the protein carbonylation in the ADR/SMAE group, indicating that SMAE could effectively scavenge ROS induced by ADR (Figure 2(a)). The protein spots with significant and meaningful changes were indicated by Arabic numerals and were subjected to a PMF analysis after using MALDI-TOF mass spectrometry.  Table 1 summarizes the detailed results obtained after using the MASCOT database searching. The individual carbonylated proteins separated by 2-DE analysis were normalized according to the intensity of the proteins. The eight targeted proteins which were uncovered by 2-DE oxyblot analysis were further dissected with the MetaCore™ software to elucidate the intracellular events and the mechanisms associated with ADR-related heart injury. The biological networks were built based on the uploaded proteins, and the biological process was appointed to each network as shown in Figure 2(b). The specific interaction pathways showed that differentially expressed proteins under the influence of ADR and SMAE administration were majorly involved in the following cellular pathways: protein folding response to unfolded proteins (*p* = 5.411 × 10^−7^), protein folding in ER and cytoplasm (*p* = 1.278 × 10^−5^), and apoptosis in mitochondria (*p* = 8.305 × 10^−2^). The *p* value demonstrates the significance of the assigned GO process on the basis of assembly size as compared with the subnetworks derived from the input protein list. According to the KEGG pathway analysis, glyoxylate and dicarboxylate metabolism, TCA cycle, pyruvate metabolism, and carbon metabolism were largely impaired due to ROS-mediated cell apoptosis and protein degradation (Figure 2(c)).

### 3.3. Evaluation of Protective Effect of SMAE against the Cytotoxicity Observed in ADR-Applied H9c2 Cells

**Figure 3:**
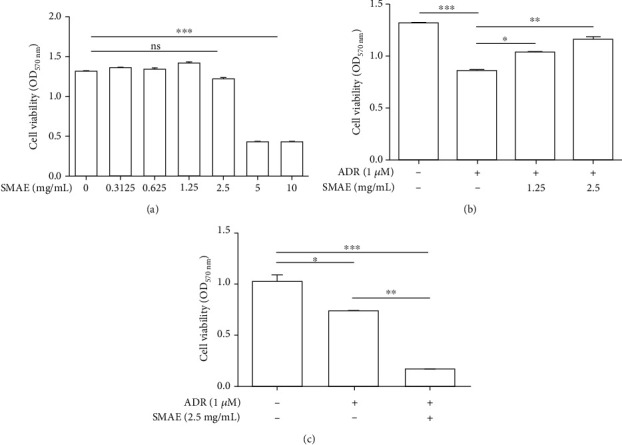
(a) Safety of SMAE extract upon H9c2 cell viability was measured by the MTT assays. H9c2 cells were treated with various concentrations of SMAE (*x*-axis). Data are mean ± SD of three independent experiments, carried out in triplicate (ns means no significance, ^∗∗∗^*p* < 0.001). (b) Viability of H9c2 cell was determined with or without 1 micro M ADR under exposure to 1.25 and 2.5 mg/mL SMAE, respectively. The quantified results were indicated by the bar chart. Results represent the mean ± SD of three independent experiments (^∗^*p* < 0.05, ^∗∗^*p* < 0.01, ^∗∗∗^*p* < 0.001). (c) Synergistic effect of ADR and SMAE upon breast cancer cell (MCF-7) was determined with or without 1 micro M ADR under exposure to 2.5 mg/mL SMAE. The quantified results were indicated by the bar chart. Results represent the mean ± SD of three independent experiments (^∗^*p* < 0.05, ^∗∗^*p* < 0.01, ^∗∗∗^*p* < 0.001).

To verify the drug safety and pharmaceutical effects of SMAE in vitro, cell viability was investigated by MTT assays. At first, H9c2 cells were treated with 0~10 mg/mL SMAE for 48 h, and cell viability was not affected under 2.5 mg/mL (Figure 3(a)). Next, SMAE application could effectively attenuate the cell death caused by the ADR exposure in a dose-dependent fashion (Figure 3(b)). The above response analysis highlighted that the optimal concentration of SMAE should be 2.5 mg/mL, while SMAE could effectively abolish the ADR-mediated cytotoxicity without causing any damage to cardiomyocytes.

### 3.4. Detection of Synergistic Cytotoxic Activities of ADR and SMAE in Breast Cancer Cells

The synergistic cytotoxic activities of ADR and SMAE were tested on the breast cancer cell line (MCF-7). The results demonstrated that SMAE could enhance the inhibitory effects of ADR, which was used to treat breast cancer. The findings suggested that the simultaneous administration of SMAE followed by chemotherapeutic agent treatment was better than the individual drug intervention (Figure 3(c)).

### 3.5. The Effect of SMAE on Cellular ROS Production and on Antioxidant Enzymes

**Figure 4:**
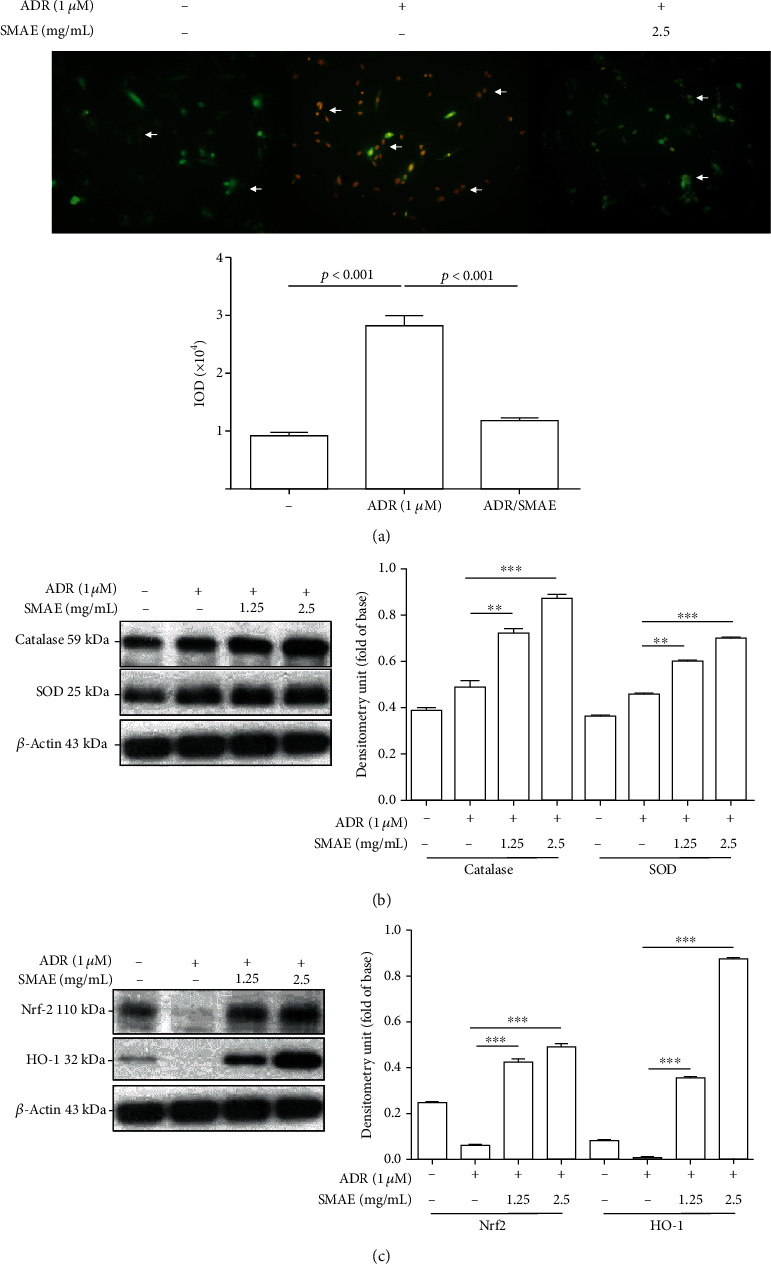
(a) H9c2 cells were incubated with or without 1 micro M ADR and 2.5 mg/mL SMAE. The DCF fluorescence signal was observed under a fluorescence microscope and demonstrated by arrows. (b) Validation of changes in protein expression after different treatments. Protein levels of catalase and SOD were determined by a Western blot analysis. (c) The expression of Nrf2 and HO-1 was evaluated with Western blot analysis. *β*-Actin was used as an internal control. The quantified results were indicated by the bar chart and represent the mean ± SD of three independent experiments. (^∗∗^*p* < 0.01, ^∗∗∗^*p* < 0.001).

Since ADR application creates an excessive amount of ROS, oxidative modification of biological molecules such as DNA and protein in the cytosol might induce consecutive apoptosis. Dichlorofluorescein (DCF) fluorescent intensity (Figure 4(a)) showed that ADR treatment promoted intracellular ROS production within 6 hours compared with the control group while treatment with 2.5 mg/mL SMAE could largely reduce the oxidative stress. We also assessed the content of oxidative stress markers, including catalase and superoxide dismutase (SOD) in the H9c2 cells. Again, treatment conducted with SMAE could significantly promote the catalase and SOD content compared with the control group as well as with the ADR-exposed subjects, thus protecting the liver against ROS damage (Figure 4(b)). To further elucidate the molecular mechanism related to the regulation of the antioxidant enzymes, Nrf2 and HO-1 which act as the predominant contributors for the activation of the antioxidant system were determined by western blot analysis. In line with this result, ADR exposure remarkably suppressed Nrf2 and HO-1 expression as compared to the control, while SMAE treatment synchronously stimulated the levels of Nrf2 and HO-1 in a dose-dependent manner (Figure 4(c)). *β*-Actin was used as a loading control. These findings suggest that SMAE could effectively repeal ADR-mediated ROS generation.

### 3.6. SMAE Modulates the Molecular Pathways of Apoptosis Induced by ADR

**Figure 5:**
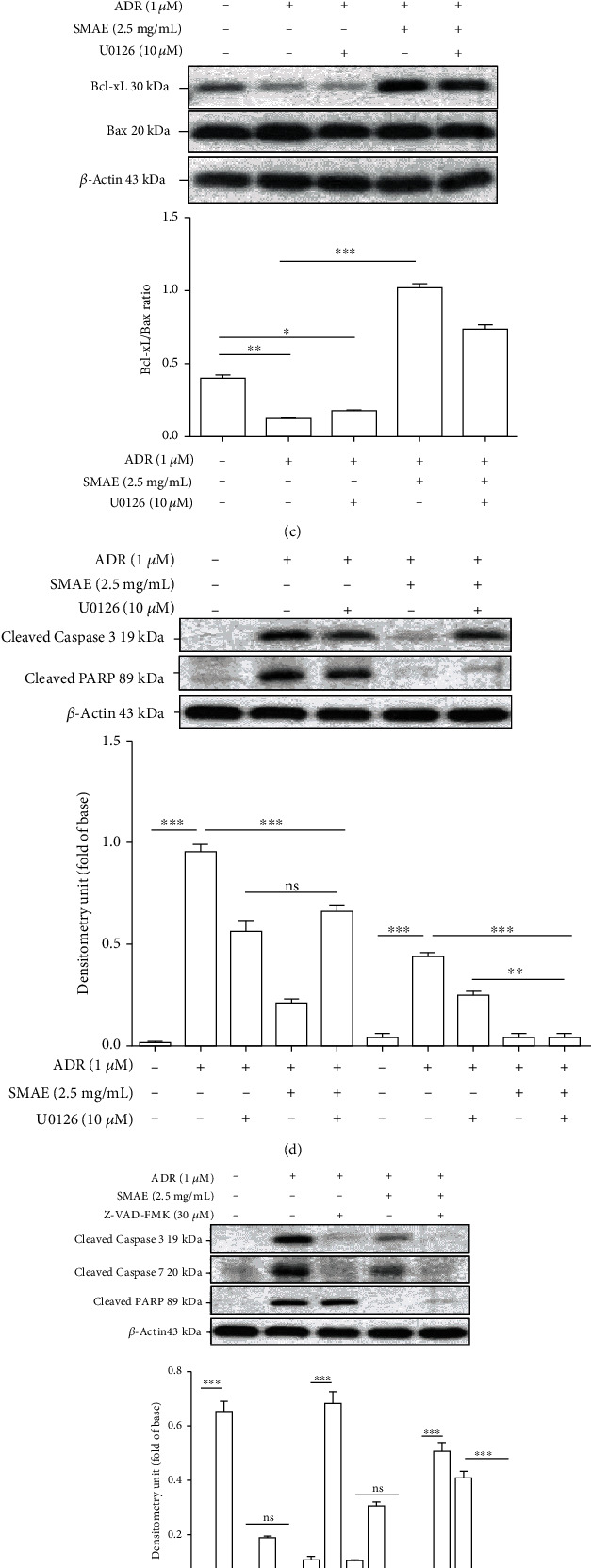
(a) Western blot analysis for phosphorylation and total protein levels of ERK1/2 and p53 with different treatments. The phosphorylation levels of ERK1/2 and p53 were normalized by total protein levels. Results represent the mean ± SD of three independent experiments (^∗∗∗^*p* < 0.001). GAPDH was applied as the loading control. (b) Cells were preincubated with or without U0126 for 0.5 h and then treated with or without ADR and SMAE for 24 h. The phosphorylation levels of ERK1/2 and p53 were normalized by total protein levels. *β*-Actin was used as an internal control. Results represent the mean ± SD of three independent experiments. (c) The protein levels of Bcl-xL and Bax with or without treatments of U0126, ADR, and SMAE were determined by western blotting assays. Density ratio of Bcl-xL over Bax was measured by densitometer, and *β*-actin was used as an internal control. The quantified results were indicated by the bar chart. (d) Cleavage of caspase-3 and PARP with or without treatments of U0126, ADR, and SMAE was determined by western blotting assays. *β*-actin was used as an internal control. The quantified results were indicated by the bar chart. (e) Cleavage of caspase-3 and PARP with or without treatments of Z-VAD-FMK (30 *μ*M), ADR, and SMAE was determined by western blotting assays. *β*-Actin was used as an internal control. The quantified results were indicated by the bar chart. (f) SMAE efficacy on protection against ADR induced cathepsin B/AIF-mediated apoptosis. The quantified results were indicated by the bar chart. Results represent the mean ± SD of three independent experiments (^∗∗∗^*p* < 0.001).

To further address the signaling events underlying the apoptotic response of H9c2 cells after exposure to ADR with or without SMAE application, we investigated the MAPK cascades which might trigger downstream signaling such as p53 pathway associated with apoptosis induction. The peaks of phosphorylated ERK1/2 and p53 protein induction were detected at 24 hours following 1 *μ*M of ADR treatment compared to the control, while exposure to SMAE dramatically inhibited ERK1/2 and p53 phosphorylation which was stimulated by ADR. Total ERK1/2 and p53 content showed no significant changes over time (Figure 5(a)). In parallel with the result mentioned above, the ERK1/2 inhibitor (10 *μ*M U0126) moderately suppressed ERK1/2 and p53 phosphorylation, which was stimulated by ADR while administration of SMAE with U0126 significantly arrested the levels of ERK1/2 and p53 phosphorylation, which reflects that SMAE could restore the ADR-caused apoptosis via ERKs/p53 signaling transduction pathway (Figure 5(b)). The increased expression of p53 is associated with Bcl-2 family genes, which elicit the cytochrome c release, activation of caspase-9 and caspase-3, and eventually cell apoptosis. Furthermore, we demonstrated that the pretreatment of SMAE with or without U0126 significantly induced the Bcl-xL/Bax ratio, which was negatively accompanied by cytochrome c release into the cytoplasm and coordinately inhibited the cell apoptosis (Figure 5(c)). In line with our findings, we measured the signaling marker proteins, caspase-3, and PARP by western blot analysis. Active forms of caspase-3 and cleaved PARP (89 kDa) were increased under the treatment of ADR, whereas the application of SMAE with or without U0126 almost completely blocked the activation of these apoptotic proteins as shown in Figure 5(d). As expected, we demonstrated that SMAE combined with or without the pan-caspase inhibitors (z-VAD-FMK) could also effectively attenuate ADR-induced apoptosis associated with a cleavage of PARP (Figure 5(e)). These results showed that the antiapoptotic effect of SMAE may be involved in modulating p53 signaling and Bcl-xL through ERK-dependent pathways. According to the abovementioned findings, a pan-caspase inhibitor could not entirely arrest ADR-induced cell death, which was manifested by cleavage of PARP. It raised the possibility that SMAE could attenuate the cardiomyotoxicity through another pathway such as the cathepsin B/apoptosis-inducing factor- (AIF-) dependent event. Western blotting results showed that ADR treatment stimulated the level of cathepsin B but significantly suppressed AIF while SMAE strongly inhibiting the expression of cathepsin B, and a corresponding increase in the levels of AIF was observed (Figure 5(f)).

## 4. Discussion

Adriamycin (ADR) is a well-established and highly effective antineoplastic agent used to treat several cancers such as breast cancer and leukemia. The clinical application of ADR has been hindered as it has caused various cardiac disorders characterized by a broad spectrum of symptoms [16, 28]. It has been reported that approximately 10% of patients exposed to ADR or its derivatives will develop cardiac complications after the cessation of chemotherapy. In this regard, the herbal medicine which has been widely used for heart protection or treatment of cancers may serve as a promising cardioprotective strategy against ADR-elicited cardiomyopathy [9, 29, 30]. Here, we have revealed the protective function and underlying molecular mechanisms of SMAE against ADR-induced cardiotoxicity *in vivo* and *in vitro*.

The *in vivo* results represented that ADR exposure initiates cardiomyopathy and stimulates specific genes involved in the apoptotic process such as the caspase-3 signaling pathway, which was verified by the reduction of cell growth under ADR treatment. SMAE exposure could ameliorate heart fibrogenesis and restore the cell survival via eliminating the expression of caspase-3 without causing any cytotoxicity to cardiomyocytes. Most importantly, SMAE utilization does not affect the efficacy of anticancer drugs but strongly suppresses the viability of cancer cells to exhibit a synergistic effect.

Growing evidence shows that ROS plays a critical role in ADR-mediated cardiocytotoxicity and the accumulation of ROS in mitochondria will finally result in cell apoptosis and will have an impact on cellular metabolism [31]. Hence, the inhibition or removal of ROS may be utilized for alleviating the heart injury caused by the ADR treatment. Particularly, SMAE comprises of several bioactive components including salvianolic acids (A and B), caffeic acid, 3,4-dihydroxyphenyl lactic acid (danshensu), and tanshinone and is confirmed to have antioxidant, anti-inflammatory, and anticancer effects [13, 32, 33]. The results have demonstrated that SMAE administration significantly restricted the production of carbonylated protein and oxidative stress induced by ADR application as shown in 2-DE oxyblot and DCF analysis. It has also been demonstrated that SMAE could induce Nrf2, which activates the Nrf2 –ARE pathway to stimulate the expression of multiple antioxidant enzymes such as HO-1. Accordingly, SMAE can prevent oxidative damage by elevating the levels of SOD and catalase in the ADR-exposed H9c2 cells. Taken together, these results imply that SMAE at least partially attenuated ADR-induced cardiomyopathy via regulating the protein carbonylation as well as the antioxidant system to relieve the ROS-mediated apoptosis.

The most striking feature observed in 2-DE oxyblot analysis is that lots of mitochondrial proteins showed significant and meaningful changes in redox state under different treatments modality. It is well-established that biomolecule oxidation is closely linked to a series of pathological events; the accumulation of redox-dependent posttranslational modification of certain key proteins would lead to ER stress and subsequent activation of the mitochondrial apoptotic pathway. The heart is one of the highest ATP-consuming organs and most of ATP is generated by the mitochondria through primarily oxidative phosphorylation [34–38]. Furthermore, network analysis has suggested that ADR-caused heart damage is majorly caused by impaired protein folding due to oxidative stress and mitochondrial dysfunction. Intertwined with this, the specific proteins including ACON, GRP75, PCCA, ENOA, KCRM, ODPA, and MDHC which are mostly associated with critical metabolic processes in mitochondria are highly oxidatively modified under ADR application, leading to cardiomyopathy, whereas SMAE treatment could remarkably abolish the oxidative stress via promoting the antioxidant capacity and therefore maintaining the normal physiological function of cardiomyocytes. These findings clearly explain that the heart is the most vulnerable organ which is greatly impacted by ADR application.

Of these proteins, aconitase (ACON) that is considered as a biomarker for oxidative stress and serves as an intramitochondrial sensor of redox status participates in the tricarboxylic acid (TCA) cycle [39]. Previous reports have indicated that the decrease in ACON activity is related to specific disorders [40]. In our study, ACON was seen to be highly carbonylated after ADR treatment and caused functional impairment, whereas oxidative modification was reversed under the SAME administration, suggesting its involvement and protective role in the management of ADR-induced toxicity occurring in cardiac mitochondria. Similarly, the PCCA protein is responsible for the formation of carboxybiotin upon the hydrolysis of ATP in the TCA cycle [41]. Enzyme function was impacted by oxidative modification caused by ADR while SMAE exposure attenuated the degree of carbonylation of PCCA to retain normal characteristics. Pyruvate dehydrogenase complex (ODPA) irreversibly decarboxylates pyruvate to acetyl coenzyme A, thereby linking glycolysis to the TCA cycle and defining a critical step in cellular bioenergetics [42]. It was noticed that ADR elicited ROS, which resulted in protein damage via carbonylation, which ultimately caused an obstacle for energy production in mitochondria. In accordance with the network analysis result, it was evident that the ADR application would interfere with particular metabolic pathways which are crucial to maintain heart functions including glyoxylate metabolism, TCA cycle, and pyruvate metabolic process. Moreover, the most oxidizable proteins are largely linked to these metabolisms. The glucose-regulated protein 75 (GRP75), belongs to the family of chaperone protein, is particularly sensitive to oxidative stress, and reduces the toxicity by oxidation itself [43]. Current evidences have indicated that the oxidation of specific chaperons could induce the apoptosis in cells to offer a checkpoint after oxidative injury. Herein, much less carbonylated GRP75 presents the possible roles of SMAE treatment in the preventive effect against ROS-mediated damage to the cardiomyocytes. In summary, increases in the production of ROS due to ADR application are significantly greater than those that can be neutralized by intracellular antioxidant defenses and result in the generation of huge oxidative stress, finally leading to apoptosis of cardiomyocytes. Of the various oxidative injuries, protein carbonylation has been considered as a potential mechanism involved in mitochondrial dysfunction, metabolic defects, and the exact contribution of carbonylation-induced dysfunction of these proteins to overall heart deficit.

Moreover, the ERK signaling pathways are proposed to be involved in NF-*κ*B transactivation during oxidative stress in myoblasts [44]. As indicated in the previous investigation, ERK phosphorylation may contribute to the initiation of p53-dependent mechanisms, which stimulate the levels of the proapoptotic Bax protein and inhibit bcl-2 expression [45]. In the current study, ERK activation followed by p53 phosphorylation, cleavage of caspase3/7, and PARP were also performed in the western blotting data. Particularly, individual administration of U0126 or Z-VAD-FMK could not entirely block the cleavage of PARP; nevertheless, pretreatment with SMAE as well as U0126 totally attenuated ADR-induced ERK1/2 phosphorylation, Bcl-xL/Bax ratio, and subsequent activation of PARP. Similarly, cotreatment of SMAE and inhibitor Z-VAD-FMK performed the powerful ability to absolutely suppress the caspase protein activation and PARP cleavage, an irreversible step toward apoptosis. These findings suggest that SMAE could protect against the ADR-caused cardiomyocyte apoptosis via multiple mechanisms except the ERK/p53/PARP signaling pathway. Consistently, SMAE treatment could also prevent ADR-caused cell death via cathepsin B-initiated partial extrinsic apoptotic cascade accompanied by the nuclear translocation of AIF, which emphasizes the important roles of mitochondria in ADR-induced cardiotoxicity as well as the protective function of SMAE.

**Figure 6:**
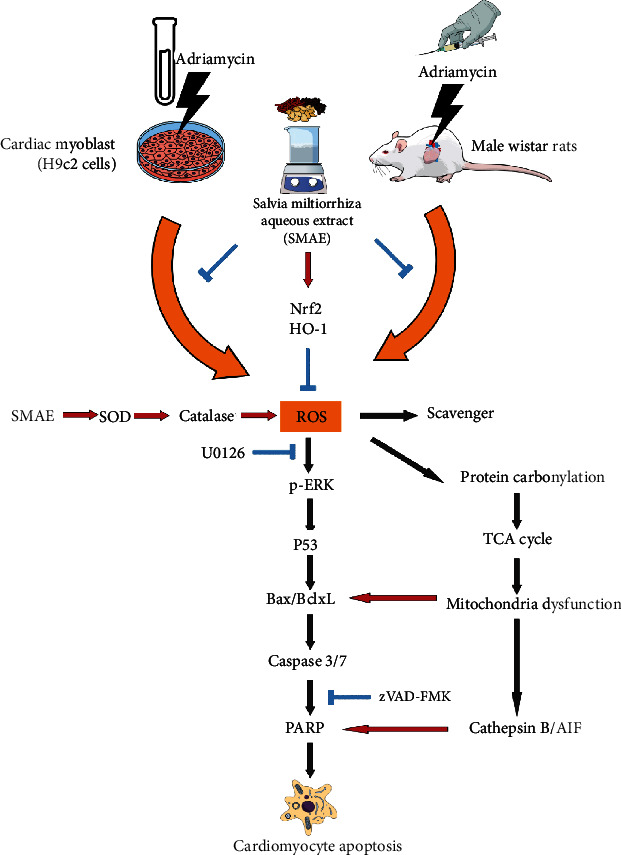
Schematic diagram of ADR-mediated cardiomyopathy through suppression of antioxidant enzymes and inducing oxidative modification of specific proteins involved in mitochondria metabolism. SMAE application could protect the heart cells against ADR damage via enhancement of antioxidant system as well as inhibition of ERK/p53- and cathepsin B/AIF-mediated apoptotic cascades.

Overall, it is clearly seen that the SMAE administration provides protection against ADR-induced cardiotoxicity via activating antioxidant enzymes and Nrf2/HO-1 signaling cascades. The apoptosis mediated by protein carbonylation and subsequent mitochondria dysfunction is diminished by SMAE application, while SMAE also inhibits ERK/p53 signaling pathway, which in turn downregulates the Bcl-xL/Bax protein ratio leading to cytochrome c release followed by caspase protein activation and PARP cleavage. In addition to the caspase-dependent pathway, the SMAE application could regulate the cathepsin B/AIF cascade and relieve the ADR-caused cell death (Figure 6). Our study offers an insight into the molecular mechanisms of ADR-related apoptosis taking place in cardiomyocytes and presents the potent clinical implication of herbal medicine which can be used for treating cancers along with the ADR administration, which is associated with the serious side effect of cardiotoxicity.

## Figures and Tables

**Table 1:** List of identified oxidation of proteins in rat heart tissue.

Spot no.	Protein name	Accession number	Mw/pI	Score (coverage)^1^	Match fragment	Location	Function
1	ACON	Q9ER34	86.121/7.87	175 (37%)	23	Mitochondrion	Catalyzes the isomerization of citrate to isocitrate via cis-aconitate.
2	GRP75	P48721	74.097/5.97	189 (49%)	24	Mitochondrion	May play a role in the control of cell proliferation and cellular aging.
3	PCCA	P14882	82.198/7.59	113 (32%)	16	Mitochondrion matrix	This is one of the 2 subunits of the biotin-dependent propionyl-CoA carboxylase (PCC), a mitochondrial enzyme involved in the catabolism of odd chain fatty acids, branched-chain amino acids isoleucine, threonine, methionine, and valine and other metabolites.
4	ENOA	P04764	47.440/6.16	171 (54%)	18	Cytoplasm or cell membrane.	Multifunctional enzyme that, as well as its role in glycolysis, plays a part in various processes such as growth control, hypoxia tolerance and allergic responses.
5	ALDH2	P11884	56.966/6.63	125 (36%)	14	Mitochondrion matrix	An aldehyde + H_2_O + NAD^+^ = a carboxylate + H^+^ + NADH
6	KCRM	P00564	43.246/6.58	78 (26%)	9	Cytoplasm.	Creatine kinase isoenzymes play a central role in energy transduction in tissues with large, fluctuating energy demands, such as skeletal muscle, heart, brain, and spermatozoa.
7	ODPA	P26284	43.883/8.49	56 (20%)	8	Mitochondrion matrix	The pyruvate dehydrogenase complex catalyzes the overall conversion of pyruvate to acetyl-CoA and CO_2_, and thereby links the glycolytic pathway to the tricarboxylic cycle.
8	MDHM	P04636	36.117/8.93	84 (42%)	12	Mitochondrion matrix	(S)-malate + NAD^+^ = H^+^ + NADH + oxaloacetate

^1^SwissProt 2020_02 (562253 sequences; 202348262 residues.

## Data Availability

(1) The Western blotting and gel image data used to support the findings of this study have been deposited in the database of Chinese medicine laboratory in Chang Gung University. (2) The Western blotting and gel image data used to support the findings of this study are included within the article. (3) The Western blotting and gel image data used to support the findings of this study are available from the corresponding author upon request.

## References

[1] Carvalho C., Santos R., Cardoso S. (2009). Doxorubicin: the good, the bad and the ugly effect. *Current Medicinal Chemistry*.

[2] Kalyanaraman B. (2020). Teaching the basics of the mechanism of doxorubicin-induced cardiotoxicity: have we been barking up the wrong tree?. *Redox Biology*.

[3] Octavia Y., Tocchetti C. G., Gabrielson K. L., Janssens S., Crijns H. J., Moens A. L. (2012). Doxorubicin-induced cardiomyopathy: from molecular mechanisms to therapeutic strategies. *Journal of Molecular and Cellular Cardiology*.

[4] Cappetta D., De Angelis A., Sapio L. (2017). Oxidative stress and cellular response to doxorubicin: a common factor in the complex milieu of anthracycline cardiotoxicity. *Oxidative Medicine and Cellular Longevity*.

[5] Renu K., Abilash V. G., Tirupathi Pichiah P. B., Arunachalam S. (2018). Molecular mechanism of doxorubicin-induced cardiomyopathy – An update. *European Journal of Pharmacology*.

[6] Schwach V., Slaats R. H., Passier R. (2020). Human pluripotent stem cell-derived cardiomyocytes for assessment of anticancer drug-induced cardiotoxicity. *Front Cardiovasc Med.*.

[7] Swain S. M., Vici P. (2004). The current and future role of dexrazoxane as a cardioprotectant in anthracycline treatment: expert panel review. *Journal of Cancer Research and Clinical Oncology*.

[8] Reichardt P., Tabone M. D., Mora J., Morland B., Jones R. L. (2018). Risk-benefit of dexrazoxane for preventing anthracycline-related cardiotoxicity: re-evaluating the European labeling. *Future Oncology*.

[9] Wang Z., Qi F., Cui Y. (2018). An update on Chinese herbal medicines as adjuvant treatment of anticancer therapeutics. *Bioscience Trends*.

[10] Tsai M. Y., Hu W. L., Lin C. C. (2017). Prescription pattern of Chinese herbal products for heart failure in Taiwan: a population-based study. *International Journal of Cardiology*.

[11] Li Z. M., Xu S. W., Liu P. Q. (2018). Salvia miltiorrhiza Burge (Danshen): a golden herbal medicine in cardiovascular therapeutics. *Acta Pharmacologica Sinica*.

[12] Ren J., Fu L., Nile S. H., Zhang J., Kai G. (2019). Salvia miltiorrhiza in treating cardiovascular diseases: a review on its pharmacological and clinical applications. *Frontiers in Pharmacology*.

[13] You J. S., Pan T. L., Lee Y. S. (2007). Protective effects of Danshen (Salvia Miltiorrhiza) on adriamycin-induced cardiac and hepatic toxicity in rats. *Phytotherapy Research*.

[14] Stěrba M., Popelová O., Vávrová A. (2013). Oxidative stress, redox signaling, and metal chelation in anthracycline cardiotoxicity and pharmacological cardioprotection. *Antioxidants & Redox Signaling*.

[15] Songbo M., Lang H., Xinyong C., Bin X., Ping Z., Liang S. (2019). Oxidative stress injury in doxorubicin-induced cardiotoxicity. *Toxicology Letters*.

[16] Wenningmann N., Knapp M., Ande A., Vaidya T. R., Ait-Oudhia S. (2019). Insights into doxorubicin-induced cardiotoxicity: molecular mechanisms, preventive strategies, and early monitoring. *Molecular Pharmacology*.

[17] Lennicke C., Rahn J., Heimer N., Lichtenfels R., Wessjohann L. A., Seliger B. (2016). Redox proteomics: methods for the identification and enrichment of redox-modified proteins and their applications. *Proteomics*.

[18] Colombo G., Garavaglia M. L., Astori E. (2019). Protein carbonylation in human bronchial epithelial cells exposed to cigarette smoke extract. *Cell Biology and Toxicology*.

[19] Wang P. W., Cheng Y. C., Hung Y. C. (2019). Red raspberry extract protects the skin against UVB-induced damage with antioxidative and anti-inflammatory properties. *Oxidative Medicine and Cellular Longevity*.

[20] Liu K., Zhang J. W., Liu X. G. (2018). Correlation between macroscopic characteristics and tissue-specific chemical profiling of the root of Salvia miltiorrhiza. *Phytomedicine*.

[21] Nakahara T., Tanimoto T., Petrov A. D., Ishikawa K., Strauss H. W., Narula J. (2018). Rat model of cardiotoxic drug-induced cardiomyopathy. *Methods in Molecular Biology*.

[22] Pan T. L., Wu T. H., Wang P. W. (2013). Functional proteomics reveals the protective effects of saffron ethanolic extract on hepatic ischemia-reperfusion injury. *Proteomics*.

[23] Fang J. Y., Wang P. W., Huang C. H., Chen M. H., Wu Y. R., Pan T. L. (2016). Skin aging caused by intrinsic or extrinsic processes characterized with functional proteomics. *Proteomics*.

[24] Wang P. W., Lin T. Y., Hung Y. C. (2019). Characterization of fibrinogen as a key modulator in patients with Wilson's diseases with functional proteomic tools. *International Journal of Molecular Sciences*.

[25] Pan T. L., Wang P. W., Huang C. C., Yeh C. T., Hu T. H., Yu J. S. (2012). Network analysis and proteomic identification of vimentin as a key regulator associated with invasion and metastasis in human hepatocellular carcinoma cells. *Journal of Proteomics*.

[26] Pan T. L., Wang P. W., Leu Y. L., Wu T. H., Wu T. S. (2012). Inhibitory effects of Scutellaria baicalensis extract on hepatic stellate cells through inducing G2/M cell cycle arrest and activating ERK-dependent apoptosis via Bax and caspase pathway. *Journal of Ethnopharmacology*.

[27] Wang P. W., Hung Y. C., Lin T. Y. (2019). Comparison of the biological impact of UVA and UVB upon the skin with functional proteomics and immunohistochemistry. *Antioxidants*.

[28] McGowan J. V., Chung R., Maulik A., Piotrowska I., Walker J. M., Yellon D. M. (2017). Anthracycline chemotherapy and cardiotoxicity. *Cardiovascular Drugs and Therapy*.

[29] Hosseini A., Sahebkar A. (2017). Reversal of doxorubicin-induced cardiotoxicity by using phytotherapy: a review. *J Pharmacopuncture.*.

[30] Huang C. H., Chang H. P., Su S. Y. (2019). Traditional Chinese medicine is associated with a decreased risk of heart failure in breast cancer patients receiving doxorubicin treatment. *Journal of Ethnopharmacology*.

[31] Berthiaume J. M., Wallace K. B. (2007). Adriamycin-induced oxidative mitochondrial cardiotoxicity. *Cell Biology and Toxicology*.

[32] Wang L., Zhang X., Chan J. Y.-W. (2016). A novel danshensu derivative prevents cardiac dysfunction and improves the chemotherapeutic efficacy of doxorubicin in breast cancer cells. *Journal of Cellular Biochemistry*.

[33] Lin Y. S., Shen Y. C., Wu C. Y. (2019). Danshen improves survival of patients with breast cancer and dihydroisotanshinone I induces ferroptosis and apoptosis of breast cancer cells. *Frontiers in Pharmacology*.

[34] Carvalho F. S., Burgeiro A., Garcia R., Moreno A. J., Carvalho R. A., Oliveira P. J. (2014). Doxorubicin-induced cardiotoxicity: from bioenergetic failure and cell death to cardiomyopathy. *Medicinal Research Reviews*.

[35] Varga Z. V., Ferdinandy P., Liaudet L., Pacher P. (2015). Drug-induced mitochondrial dysfunction and cardiotoxicity. *American Journal of Physiology. Heart and Circulatory Physiology*.

[36] Akhmedov A. T., Rybin V., Marín-García J. (2015). Mitochondrial oxidative metabolism and uncoupling proteins in the failing heart. *Heart Failure Reviews*.

[37] Sheeran F. L., Pepe S. (2017). Mitochondrial bioenergetics and dysfunction in failing heart. *Advances in Experimental Medicine and Biology*.

[38] Ichihara S., Suzuki Y., Chang J. (2017). Involvement of oxidative modification of proteins related to ATP synthesis in the left ventricles of hamsters with cardiomyopathy. *Scientific Reports*.

[39] Lushchak O. V., Piroddi M., Galli F., Lushchak V. I. (2013). Aconitase post-translational modification as a key in linkage between Krebs cycle, iron homeostasis, redox signaling, and metabolism of reactive oxygen species. *Redox Report*.

[40] Talib J., Davies M. J. (2016). Exposure of aconitase to smoking-related oxidants results in iron loss and increased iron response protein-1 activity: potential mechanisms for iron accumulation in human arterial cells. *Journal of Biological Inorganic Chemistry*.

[41] Wongkittichote P., Ah Mew N., Chapman K. A. (2017). Propionyl-CoA carboxylase - a review. *Molecular Genetics and Metabolism*.

[42] Sun W., Liu Q., Leng J., Zheng Y., Li J. (2015). The role of pyruvate dehydrogenase complex in cardiovascular diseases. *Life Sciences*.

[43] Hung Y. C., Wang P. W., Pan T. L., Bazylak G., Leu Y. L. (2009). Proteomic screening of antioxidant effects exhibited by radix Salvia miltiorrhiza aqueous extract in cultured rat aortic smooth muscle cells under homocysteine treatment. *Journal of Ethnopharmacology*.

[44] Abdelhamid G., El-Kadi A. O. S. (2015). Buthionine sulfoximine, an inhibitor of glutathione biosynthesis, induces expression of soluble epoxide hydrolase and markers of cellular hypertrophy in a rat cardiomyoblast cell line: roles of the NF-*κ*B and MAPK signaling pathways. *Free Radical Biology & Medicine*.

[45] Liu J., Mao W., Ding B., Liang C. S. (2008). ERKs/p 53 signal transduction pathway is involved in doxorubicin-induced apoptosis in H9c2 cells and cardiomyocytes. *American Journal of Physiology. Heart and Circulatory Physiology*.

